# Recurrent Peri-Clitoral Abscess with Positive *Actinomyces turicensis* Culture

**DOI:** 10.1155/2023/9912910

**Published:** 2023-07-13

**Authors:** Diana Palacios, Cristina Wallace Huff

**Affiliations:** ^1^Department of Obstetrics and Gynecology, Baylor College of Medicine in Houston, Houston, TX, USA; ^2^Department of Obstetrics and Gynecology, University of Texas Health Science in San Antonio, San Antonio, TX, USA

## Abstract

A peri-clitoral abscess is a condition that is seldom encountered in practice and is found scarcely in the literature. The cause of spontaneous peri-clitoral abscess not associated with female circumcision/genital mutilation is generally unknown. Additionally, there have been no case reports of positive *Actinomyces* culture at the time of drainage of a peri-clitoral abscess. This case outlines a 42-year-old female with a spontaneous peri-clitoral abscess. The abscess was initially treated with incision and drainage (I&D) and antibiotics, but it later reoccurred necessitating a second I&D with bedside marsupialization and antibiotics targeted at *Actinomyces*, which grew on the culture after primary I&D.

## 1. Introduction

A spontaneous peri-clitoral abscess is an uncommon gynecological condition with limited reports in the literature. Typically, patients present with a painful, inflamed fluctuant area surrounding the clitoris. While some cases are associated with a pilonidal disease or genital trauma, such as female circumcision/genital mutilation, others occur spontaneously without an identifiable cause [1]. The etiology of spontaneous peri-clitoral abscesses remains unclear due to the limited number of cases reported. Treatment options include medical management with antibiotics alone or in combination with surgical interventions, such as incision and drainage (I&D) with marsupialization, which have a low success rate in preventing recurrence [1].

## 2. Case Presentation

A 42-year-old G5P3023 female presented to the clinic, reporting a recurrent mass on her right vulva near her clitoris for six months. The patient reports that the mass would become inflamed, then resolve spontaneously over several weeks, draining a clear fluid each time. She had a history of three previous vaginal deliveries but no history of peri-urethral, or peri-clitoral tears at the time of her deliveries. Her most recent delivery was three years before the initial appearance of the abscess and was non-operative with no documented tears. She denied a history of trauma to the area. On initial presentation, she had an acute episode of the peri-clitoral mass with inflammation and mild bloody drainage, all of which started about two weeks before. She reported that the mass was slightly tender to palpation and bothersome. She had a Kyleena intrauterine device (IUD) for contraception, which had been in place for three years. She had no fever or chills and denied abdominal pain, pelvic pain, urinary symptoms, and vaginal discharge. She had normal vital signs. On physical examination, she had a 2 cm × 1 cm area of inflammation with erythema and fluctuance about 0.5 cm lateral to the clitoral hood on the right side. Non-odorous yellow fluid drained from the abscess. An I&D of the abscess was performed in the clinic through a 1 cm vertical skin incision along the area of inflammation, and aerobic and anaerobic cultures were sent for further evaluation. There was no evidence of uterine infection, so the IUD was left in place. The patient was started on a 10-day oral course of trimethoprim/sulfamethoxazole. On day 5 after the procedure, the culture returned positive for *Actinomyces turicensis*. The patient's antibiotic regimen was changed to a 14-day course of amoxicillin to target *Actinomyces*. The patient reported adherence to the amoxicillin as prescribed. She was then seen in the clinic two weeks after the initial I&D. At that time, the lesion looked well healed on physical examination, and the patient reported improvement in symptoms. The patient again reported adherence to the antibiotics and reported having 5 days left of the 14-day course of amoxicillin.

**Figure 1:**
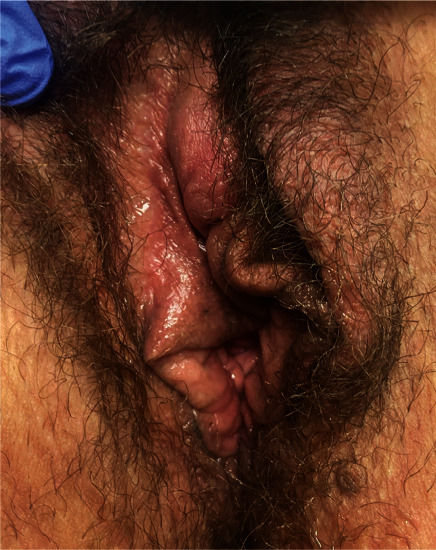
Before marsupialization.

**Figure 2:**
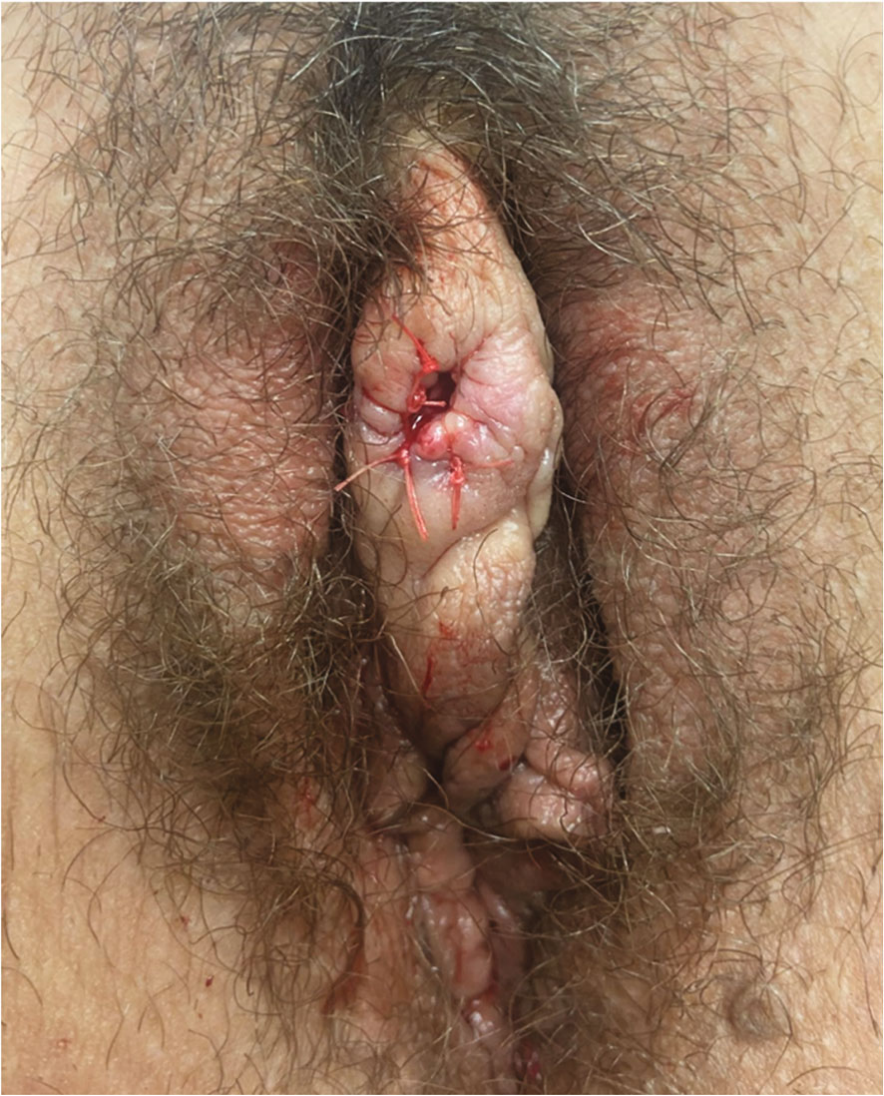
Immediately after marsupialization.

**Figure 3:**
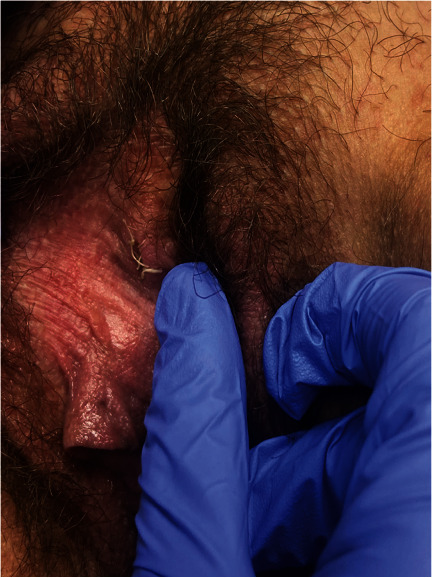
Two weeks after marsupialization.

One month after the follow-up visit, the patient was seen in the clinic for recurrence of the peri-clitoral abscess in the same location as seen in Figure 1. Again, there was no abdominal or pelvic pain, vaginal discharge, or fever. The patient was not on antibiotics at this time but reported having finished the 14 days of oral amoxicillin as prescribed about three weeks before. As seen in Figure 2, I&D with marsupialization of the abscess was performed in the clinic with local anesthesia. The cyst was probed to exclude a sinus tract; however, no sinus tract was discovered. A repeat culture of the abscess fluid was ordered. Based on the previous culture, the decision was made to remove the IUD, and the patient was started on a six-week course of ampicillin. Aerobic cultures for the second I&D returned negative for all pathogens. Anaerobic cultures were not performed due to laboratory cancellation. Two weeks later, the patient was seen again in the clinic. As seen in Figure 3, there was a slight bulge in the right-sided peri-clitoral region, in the same area previously affected, without any pain or discharge from the site. The patient reported adherence to the ampicillin as prescribed. The patient was counseled to continue the antibiotic course and to return to the clinic if the abscess returned or if symptoms worsened. During her six-week follow-up, the patient mentioned experiencing some clear, odorless drainage from the wound a few weeks after the I&D with the marsupialization procedure. However, she reported having no pain or inflammation in the area and attributed the episode to normal healing. She also reported completion of the ampicillin. Upon physical examination, the sutures were found to be fully healed with no redness or inflammation in the area. No further treatment was deemed necessary, and the patient was advised to return to the clinic if symptoms reoccurred. As of the 6-month mark after the I&D with marsupialization, the patient has not reported any further recurrence of symptoms.

## 3. Discussion

A peri-clitoral abscess is a rare condition in which an abscess forms in the tissue surrounding the clitoris. Like most peri-clitoral abscesses described in the literature, it is unclear what led to the development of this patient's abscess. Etiologies of peri-clitoral abscesses include infected pilonidal cysts and trauma; however, most are idiopathic. Pilonidal cyst abscesses are due to infection of the pilonidal tract leading to abscess formation [2–4]. Exploration of these abscesses for sinus tracts or hair follicles within or near the abscesses indicates this etiology [5]. Upon exploration and marsupialization of this patient's abscess, no sinus tract was found, making pilonidal disease an unlikely etiology. Although a physical examination can sometimes make it challenging to confirm the presence of a sinus tract, the cyst in this case was thoroughly probed and examined. However, no sinus tract was found, which helped to rule out its potential role in causing the recurrence. Additionally, the patient had no history of obvious trauma to the area. However, it is possible that there was minor trauma from sexual activity or undetected trauma during her most recent childbirth 3 years before the appearance of this lesion. At the time of her last delivery, the patient had no documented tears and no operative delivery. Currently, this patient's abscess is classified as idiopathic due to the lack of a sinus tract upon exploration and the lack of identified trauma to the area, which is the most common etiology for peri-clitoral abscesses.

The first culture of the abscess fluid grew *A. turicensis*, an uncommon pathogen in the female reproductive tract. Microorganism cultures from peri-clitoral abscesses noted in the literature include *Staphylococcus aureus*, *Staphylococcus epidermidis*, *Peptostreptococcus*, *Bacteroides fragilis*, diphtheroids, Coagulase-positive *Staphylococcus*, *Streptococcus bovis*, and *Streptococcus anginosus.* However, no previous case reports of peri-clitoral abscesses mention positive *Actinomyces* spp. cultures. Interestingly, *Actinomyces* is detected in 8–20% of IUD users [6]. This patient had an IUD that was inserted approximately three years before the initial abscess. *Actinomyces* spp. is incidentally detected in up to 25% of these patients and typically does not result in infection [6]. Studies have shown that incidentally discovered *Actinomyces* in asymptomatic patients have minimal significance and do not require treatment or removal of the IUD [6]. Pelvic actinomycosis associated with IUDs is characterized by fever, weight loss, and abdominal pain, and its treatment involves removal of the IUD, potential surgical debridement, and a minimum of six weeks of antibiotic therapy [7]. However, the patient, in this case, did not experience abdominal pain, fever, or chills, and her sole complaint was the localized infection, making pelvic actinomycosis highly improbable. Following the patient's first I&D, a joint decision was made to leave the IUD in place given that the patient had no symptoms of pelvic actinomycosis, and she desired long-term contraception. It is worth noting that we were unaware of the presence of *A. turicensis* at that time, and this information was not considered when deciding to retain the IUD. Following abscess recurrence, and with the awareness of the literature indicating the presence of *Actinomyces* in up to 20% of IUD users, a cautious approach was adopted, and the decision was made to remove the IUD to prevent potential re-seeding of the abscess [6].

It is important to note that although our team requested both aerobic and anaerobic cultures at the time of recurrence, the laboratory cancelled the orders for anaerobic culture and Gram stain. These tests could have potentially detected the presence of *Actinomyces*. This indicates that the *Actinomyces* infection may have persisted at that point but went unnoticed due to inadequate testing. Routine aerobic cultures may not always provide optimal conditions for the growth and identification of anaerobic bacteria, highlighting the necessity of conducting anaerobic cultures for abscess fluid analysis. Additionally, performing a Gram stain on the purulent abscess fluid would have allowed visualization of bacterial forms, including *Actinomyces* spp., even if they were nonviable due to exposure to oxygen. Incorporating a Gram stain would have been beneficial in identifying a broader range of bacteria, including anaerobic species, that might have been missed by a routine aerobic culture. This case serves as a reminder of the significance of utilizing multiple diagnostic tools, such as the Gram stain, to our advantage as clinicians in the diagnosis and management of anaerobic infections that are prone to being undetected in routine cultures. Furthermore, it emphasizes the importance of effective interdepartmental communication as we handle complex cases.

Given the incomplete results of the second culture, we have considered several possibilities regarding the *Actinomyces* identified in the initial culture. The results of the abscess drainage fluid culture obtained during the removal of the IUD and I&D with marsupialization did not show the growth of any pathogens, which could potentially be attributed to the 14-day administration of amoxicillin before the IUD removal. Another possibility is that, since only aerobic cultures were conducted, the *Actinomyces* infection might have persisted at that time but went undetected during subsequent recurrences, possibly due to reseeding from the IUD. Alternatively, it is plausible that the abscess in this patient is unrelated to the IUD and instead originated from the introduction of fecal matter, which is known to harbor *Actinomyces* [6]. The nature of the abscess, whether it was caused by *Actinomyces* infection or if the presence of *Actinomyces* was incidental and insignificant, remains unclear. It is possible that the infection causing the abscess could be due to a different pathogen not discovered in our tests. It is also possible that the initial presence of *A. turicensis* in the culture could have been due to sample contamination.

*Actinomyces* infections have been documented as very difficult to treat. They are typically treated using high-dose penicillin, ceftriaxone, or amoxicillin but symptoms often recur regardless [8]. Oral treatment is usually continued for 1–2 months for mild disease and 6–12 months for severe disease [8]. Severe disease is characterized by significant purulence and formation of fistulous tracts, which this patient did not have [8]. The patient presented in this case was put on amoxicillin to which she responded well evidenced by improving clinical signs and the absence of bacteria in subsequent cultures. She has later put on a longer course of ampicillin after symptom recurrence. In retrospect, it is possible that the patient's risk of recurrence could have been lowered if a high-dose penicillin or amoxicillin, known for its higher bioavailability, had been chosen for subsequent treatment at the time of recurrence. This is because the later testing conducted was less extensive, and the possibility of a remaining *Actinomyces* spp. infection was not completely ruled out. Although penicillin and amoxicillin are the recommended treatments for actinomycosis, the six-week course of ampicillin administered during the resurgence of symptoms still provided coverage against *Actinomyces* spp. It is challenging to determine the exact impact as the abscess resolved following marsupialization and treatment with ampicillin.

There are no concrete guidelines on how peri-clitoral abscesses should be treated initially. In the literature, patients are treated based on presenting symptomatology. Generally, treatment is based on the severity of symptoms and the comfort level of the provider but often reoccurs regardless of the treatment method [1]. Typically, less invasive methods are attempted first, especially if the patient is prepubescent or adolescent. Surgical drainage under total anesthesia or conscious sedation may also be attempted as first-line treatment in these cases. In adults, I&D is often used as a first-line treatment; however, conservative management with only oral antibiotics has also been used successfully. Marsupialization has been used as a method in recurrent cases, which allows for exploration of the area and removal of possible nidus for infection, which sometimes can include hair and hair follicles [2]. However, it increases risks, such as damage to the clitoris. In this case, the initial treatment approach was a combination of I&D and a 10-day course of antibiotics. However, this approach was modified after the culture results showed the presence of bacteria that were resistant to the prescribed antibiotics. An additional 14-day course of antibiotics was given, followed by six weeks of targeted antibiotic therapy after the abscess recurred. Notably, there was a marked improvement in symptoms following the marsupialization procedure.

## 4. Conclusion

In summary, peri-clitoral abscesses remain a rare gynecological condition with limited knowledge of their etiology and treatment guidelines. This case report highlights the intricacies of diagnosing and managing peri-clitoral abscesses, particularly in the context of an atypical bacterial culture. Subsequent research is required to establish potential causative factors and develop therapeutic strategies aimed at minimizing the high recurrence rates observed in literature and encountered in this case.

## Additional Points

*Key Learning Points.* The key learning points are as follows.

- The peri-clitoral abscess should be considered in the differential diagnosis for patients presenting with a mass in the anterior vulva or clitoral region.
- Treatment options for peri-clitoral abscesses include antibiotic therapy, I&D, and marsupialization. When selecting a management approach, it is important to take into account the patient's age, pain tolerance, risk of clitoral injury, severity of symptoms, and patient preference.
- Peri-clitoral abscesses have a high recurrence rate with all treatment options described in the literature, including surgical approaches, such as marsupialization. Therefore, discussing the prognosis with patients and managing their expectations are crucial.
